# Mesenteric Lymph Duct Drainage Attenuates Lung Inflammatory Injury and Inhibits Endothelial Cell Apoptosis in Septic Rats

**DOI:** 10.1155/2020/3049302

**Published:** 2020-10-21

**Authors:** Yongjun Liu, Chuanxi Chen, Qing Sun, Huadong Sun, Ning Liu, Qier Liu, Jie Ma, Pingping Wang, Chunlin Hu, Jianfeng Wu, Bin Ouyang, Juan Chen, Minying Chen, Xiangdong Guan

**Affiliations:** ^1^Department of Critical Care Medicine, The First Affiliated Hospital, Sun Yat-Sen University, Guangzhou, Guangdong 510080, China; ^2^Department of Pathology, Sun Yat-Sen Memorial Hospital, Sun Yat-sen University, Guangzhou, Guangdong 510120, China; ^3^Department of Molecular and Cell Biology, College of Liberal Arts and Sciences, University of Connecticut, Storrs, Connecticut 06269, USA; ^4^Department of Emergency, The First Affiliated Hospital, Sun Yat-Sen University, Guangzhou, Guangdong 510080, China

## Abstract

The present study was to investigate the effect of mesenteric lymph duct drainage on lung inflammatory response, histological alteration, and endothelial cell apoptosis in septic rats. Animals were randomly assigned into four groups: control, sham surgery, sepsis, and sepsis plus mesenteric lymph drainage. We used the colon ascendens stent peritonitis (CASP) procedure to induce the septic model in rats, and mesenteric lymph drainage was performed with a polyethylene (PE) catheter inserted into mesenteric lymphatic. The animals were sacrificed at the end of CASP in 6 h. The mRNA expression levels of inflammatory mediators were measured by qPCR, and the histologic damage were evaluated by the pathological score method. It was found that mesenteric lymph drainage significantly reduced the expression of TNF-*α*, IL-1*β*, and IL-6 mRNA in the lung. Pulmonary interstitial edema and infiltration of inflammatory cells were alleviated by mesenteric lymph drainage. Moreover, increased mRNA levels of TNF-*α*, IL-1*β*, IL-6 mRNA, and apoptotic rate were observed in PMVECs treated with septic lymph. These results indicate that mesenteric lymph duct drainage significantly attenuated lung inflammatory injury by decreasing the expression of pivotal inflammatory mediators and inhibiting endothelial apoptosis to preserve the pulmonary barrier function in septic rats.

## 1. Introduction

In the pathogenesis of multiple organ dysfunction syndrome (MODS), the gut has been identified as the central organ [1–3]. It has been recognized that splanchnic circulation is particularly vulnerable to hypoperfusion status, such as severe trauma, infection, and burn, which contributes to the loss of gut barrier function and ensuing translocation of bacteria and endotoxins from the gut to the portal blood [4]. Through the portal system, gut-derived pathogenic factors can migrate to other organs, an event that plays a crucial role in triggering, perpetuating, and exacerbating the hypermetabolic and immunoinflammatory responses observed after severe trauma and shock.

Nevertheless, bacteria/endotoxin translocations were not observed in the portal blood of MODS patients [5]. Moreover, the abovementioned translocation pathway for gut-derived factors does not adequately explain the tendency of the lung to be identified as the starting organ of MODS. These views are supported by studies in rats subjected to trauma (laparotomy) and hemorrhagic shock, which have shown that the gut-derived inflammatory factors contained in the mesenteric lymph, rather than those in the portal vein, were the cause of lung injury [6]. It has also been observed that pulmonary injury induced by hemorrhagic shock was alleviated by ligation of the mesenteric lymph duct [7, 8]. Mesenteric lymph enters systemic circulation via the thoracic duct, which empties into the subclavian vein, thus making the lung the first organ exposed to mesenteric lymph. It is therefore not surprising that gut injury is implicated in the pathogenesis of lung injury [9, 10]. In trauma-hemorrhagic shock models, mesenteric lymph was found to induce injury to pulmonary microvascular endothelial cells (PMVEC) and increase lung permeability [11]. This mode of injury to the lung, which is caused by gut-derived inflammatory factors contained in the mesenteric lymph, has been named the “gut-lung axis” [12, 13].

In clinical practice, sepsis most commonly affects the lung and frequently causes acute lung injury (ALI) as well as acute respiratory distress syndrome (ARDS) via the pathway of the gut-lung axis [14]. It is uncertain whether gut-derived inflammatory mediators aggravate damage of the lung by way of the mesenteric lymph entrance into the pulmonary blood flow in septic shock. We hypothesized that lung injury in sepsis is partly due to endotoxin and inflammatory mediators via mesenteric lymph produced from the intestine. We reason that lung injury could be mitigated by broken the gut-lung axis via mesenteric lymph drainage (MLD). In this study, we aimed to investigate the pathological mechanism of septic mesenteric lymph in sepsis-induced ALI. We employed an in vivo sepsis model using rats induced by the CASP procedure and then performed mesenteric lymph drainage to verify whether the “gut-lung axis” exists in the septic rats. We studied the effect of MLD on the cytokine expression and histological alteration of the intestine, liver, and lung in septic rats. Moreover, in vitro study, PMVECs were treated with septic lymph to observe its effect on the cytokine expression and apoptosis.

## 2. Materials and Methods

### 2.1. Experimental Animals

Animal study was approved by the Sun Yat-sen University Medical School Animal Care and Ethics Committee on Research Animal Care and conducted in compliance with the guidelines of the Committee on Care and Use of Laboratory Animal Resources.

Adult male SD rats (weight 330–380 g) which were purchased from the Guangdong Medical Experimental Animal Center (Guangzhou, China) were randomly assigned to four groups (*n* = 8 per group): control, sham surgery, sepsis, and sepsis plus mesenteric lymph drainage (sepsis+MLD). An additional eight rats were used for the acquisition of normal mesenteric lymph. The rats were kept in a temperature-controlled environment with a normal light-dark cycle and free access to food and water. Prior to experiments, rats were subjected to an overnight fast but allowed access to drinking water.

### 2.2. Experimental Protocol for Collection of Mesenteric Lymph and Induction of the CASP Sepsis Model

To measure the animal's arterial blood pressure during the experiments continuously, the femoral artery was cannulated with polyethylene (PE) tubing. Next, aseptic cannulation of the internal jugular vein was inserted with a 50-guage-silicone catheter from which blood sample was obtained into a syringe containing 10 units of heparin suspended in 0.3 ml of 0.9% normal saline solution to prevent clotting. After laparotomy, a PE50 catheter was placed into the efferent lymph duct, draining the mesenteric lymph node complex. Following this preparation, either CASP surgery or sham surgery was performed [7, 15].

The method of CASP was induced as described previously [16–18]. In the shame group, a sham surgery was performed, in which the stent was not introduced into the intestinal lumen, but instead, affixed antimesenteric onto the intestinal wall externally.

In the studies, herein, septic and normal lymph were collected within 6 hours following either the CASP or sham surgery and tested at a 10% (vol/vol) concentration as previously described [15]. The blood volume of a rat is about 6% of its body weight, so that a 350 g rat would be considered to have a blood volume of 21 ml. During the period of surgical drainage, lymph production is approximately 0.4 mL/h yielding 2.4 ml over 6 hours. In our previous study, pulmonary inflammatory injury became apparent 6 hours following the CASP surgery [19]. The 2.4 ml of lymph produced would represent about 10% of the 21 ml blood volume of the rat. Accordingly, testing septic lymph at a 10% vol/vol concentration in vitro seemed clinically and biologically reasonable.

### 2.3. Histologic Measurement of Lung, Intestine, and Liver Injury

The lung, intestine, and liver tissues were harvested and immediately fixed with 4% paraformaldehyde, embedded in paraffin and cut in 4 *μ*m sections, and then stained with hematoxylin and eosin. The slides were read under a standard light microscope and scored for injury according to histologic grading scales for each tissue type [20, 21].

### 2.4. Myeloperoxidase Activity Assay

The myeloperoxidase (MPO) activity was analyzed as a marker of total neutrophil sequestration. The MPO activity in the supernatants of tissue homogenate supernatant was determined by measuring the H_2_O_2_-mediated oxidation of *o*-dianisidine hydrochloride as described previously [22]. The MPO activity was expressed as U/g tissue.

### 2.5. Cocultivation of Rat Primary Pulmonary Microvascular Endothelial Cells (PMVECs) with Mesenteric Lymph

PMVECs were generated and grown in endothelial growth medium (Lonza) containing 2% FBS. Only cells from passages 3 and 4 were used. Normal and septic mesenteric lymph were mixed with medium (at a 10% vol/vol concentration) to assess the impact of septic mesenteric lymph on PMVECs. To evaluate the potential cytotoxicity of mesenteric lymph, MTT assays were performed to assess cell viability. After a 12-hour coincubation, the morphological characteristics of the PMVECs were observed with an inverted microscope. TNF- *α* and IL-1 mRNA of PMVECs were measured by real-time PCR, and apoptosis of PMVECs was measured with flow cytometry.

### 2.6. Real-Time PCR Detection of TNF-*α*, IL-1*β*, and IL-6mRNA in the Intestine, Liver, and Lung, and as well as in PMVECs

Total RNA was extracted with TRIzol reagent (Invitrogen, USA) from the homogenized tissue. cDNA synthesis was performed using the PrimeScript™ RT reagent Kit (TakaRa, Japan) according to the manufacturer's guide. The cDNA was amplified by real-time PCR using primers for TNF-*α*, IL-1*β*, or IL-6 with *β*-actin serving as the internal control.

Amplification steps were as follows: predenaturation at 95°C for 10 min, denaturation at 95°C for 5 sec, annealing at 60°C for 30 sec, and polymerizing at 72°C for 15 sec. The 5′- and 3′-primers for TNF-*α* were CAT GGA TCT CAA AGA CAA CCA A and CTC CTG GTA TGA AAT GGC AAA T, respectively. The 5′- and 3′-primers for IL-1*β* were CTT CAA ATC TCA CAG CAG CAT C and GCT GTC TAA TGG GAA CAT CAC A, respectively. The 5′- and 3′-primers for IL-6 were TTC TCT CCG CAA GAG ACT TCC and TCT CCT CTC CGG ACT TGT GAA, respectively. The 5′- and 3′-primers for *β*-actin were CAC CCG CGA GTA CAA CCT TC and CCC ATA CCC ACC ATC ACA CC, respectively. The real-time PCR data was analyzed and presented as relative gene expression, using the comparative C_T_ method, also commonly referred to as the 2^-△△CT^ method [23].

### 2.7. Assay of TNF-*α*, IL-1*β*, and IL-6 in Mesenteric Lymph and Blood

The concentrations of TNF-*α*, IL-1*β*, and IL-6 in mesenteric lymph and blood were measured using a commercially available enzyme-linked immunosorbent assay (ELISA) kit (Invitrogen, USA), according to the manufacturers' instructions.

### 2.8. Apoptosis Analysis

Lung tissue specimens blocked by paraffin were cut in 4-*μ*m-thick sections and stained for TdT-mediated dUTP nick end labeling- (TUNEL-) positive cells using the VasoTACS In Situ Apoptosis Detection Kit (Trevigen, Gaithersburg, MD). Using a standard light microscope, 200 high-power fields (hpf) for each animal were observed, and the number of TUNEL-positive cells was counted in a blinded fashion.

For in vitro studies, apoptosis of PMVECs was detected using Annexin V-FITC/propidium iodide (PI) staining. Briefly, 1 × 10^6^ cells per sample were washed twice with ice-cold PBS and then, 5 *μ*l of Annexin V-FITC and 10 *μ*l of PI (Sigma-Aldrich, USA) were added to 100 *μ*l of the cell suspension. This was followed by an incubation period of 15 min at room temperature in the dark, after which 400 *μ*l of binding buffer was added. For each sample, the mixture was filtered using a 300-mesh nylon net prior to flow cytometry (FCM) evaluation with an EPICS XL (Beckman Coulter, USA) cytometer. EXPO32 ADC analysis software was used to analyze the data.

### 2.9. Analysis of Pulmonary Vascular Permeability

Lung vascular permeability was measured with the Evans blue dye technique at the end of the 6-hour CASP sepsis period, as previously described [6]. The recovered bronchoalveolar lavage fluid was assayed for dye concentration spectrophotometrically at 620 nm. The concentration of dye was then plotted on a standard curve, and the percentage of dye in the bronchoalveolar lavage fluid relative to the plasma concentration was determined.

### 2.10. Statistical Analysis

The SPSS 19.0 software (SPSS Inc., Chicago, IL) was used for data analysis. Intergroup differences were evaluated using one-way analysis of variance (ANOVA). For the pathological scores, the differences were evaluated using the Kruskal-Wallis nonparametric ANOVA test. *p* < 0.05 was considered statistically significant.

## 3. Results

### 3.1. Histomorphology of the Lung, Intestine, and Liver

**Figure 1:**
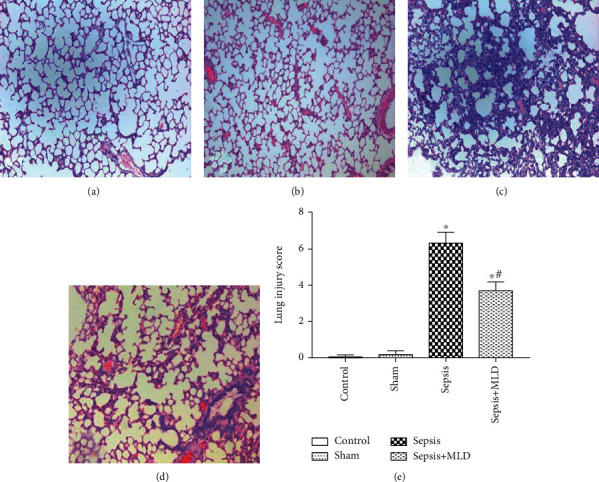
Morphologic changes of lung and evaluation of lung injury under light microscopy (magnification, ×100). There was no evidence of lung injury in the control group (a) and sham surgery group (b). In contrast, evidence of increased interstitial edema and inflammatory dell infiltration was found in the sepsis group (c). The injury degree of interstitial edema and inflammatory cell infiltration was ameliorated in the sepsis+MLD group (d). The data of the lung injury score are expressed as mean ± SE, *n* = 8 (e). Results were compared by one-way ANOVA with Student-Newman-Keul's posthoc test. ^∗^*p* < 0.05*vs.* control group; ^#^*p* < 0.05 vs. sepsis group.

Both pulmonary interstitial edema and inflammatory cell infiltration were present in the lungs of the sepsis group, but the degree of injury was ameliorated in the Sepsi s+MLD group (Figures 1(c) and 1(d)). There was no evidence of lung injury in either the control group or sham surgery group (Figures 1(a) and 1(b)). The grade of lung injury, as assessed by the lung injury grading score, was significantly decreased in the sepsis+MLD group, when compared to that in the sepsis group (*p* < 0.05) (Figure 1(e)). However, despite the significant difference in the grade of lung injury between the sepsis+MLD and control groups, the histomorphological lesions in the lungs of the CASP sepsis model-induced groups were attenuated by mesenteric lymph drainage.

**Figure 2:**
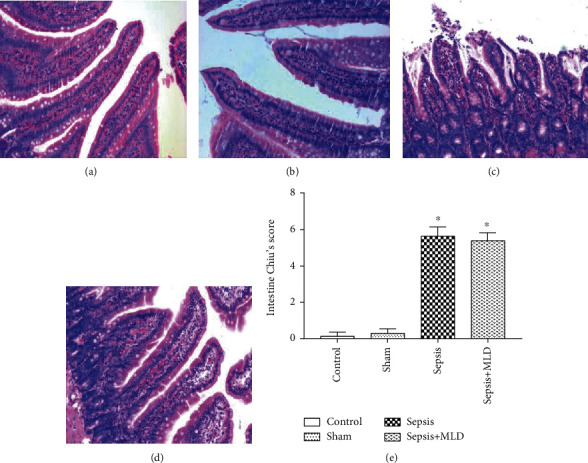
Morphologic changes of intestinal mucosa and evaluation of gut injury with Chiu's scores under light microscopy (magnification, ×200). The control group (a) and the sham operation group (b) had normal villi and glands. By contrast, severe edemas of mucosal villi accompanied with severe intestinal gland injury were observed in the sepsis group. In addition, a large number of intestinal villi disintegrated, the gap of epithelial cells increased, and severe hemorrhage was present, indicative of severe mucosal damage in the sepsis group (c). The putrescence and desquamation of epithelial cells in the intestinal mucosa were attenuated, but mucosal sloughing could be seen at villi tips, and the gap between epithelial cells increased slightly in the sepsis+MLD group (d). The data of Chiu's scores were expressed as mean ± SE, *n* = 8 (e). Results were compared by one-way ANOVA with Student-Newman-Keul's posthoc test. ^∗^*p* < 0.05 vs. control group.

**Figure 3:**
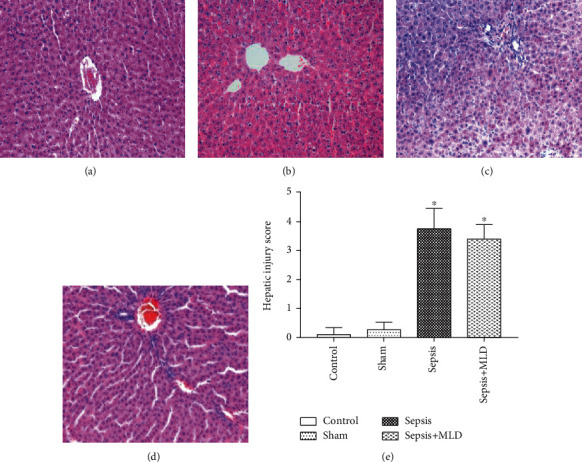
Morphologic changes of liver and evaluation of liver injury under light microscopy (magnification, ×200). There was no apparent change of liver in the control group (a) and sham surgery group (b). In the sepsis group, swollen hepatocyte, raritas, or lucency kytoplasm were observed. In additional, dilated sinus hepaticus and lymphocyte infiltration in converged tube were also observed in the sepsis group (c). The injury degree of sinus hepaticus dilation and inflammatory cell infiltration was improved in the sepsis+MLD group (d). The data of the liver injury score are expressed as mean ± SE, *n* = 8 (e). Results were compared by one-way ANOVA with Student-Newman-Keul's posthoc test. ^∗^*p* < 0.05 vs. control group.

Changes in the intestinal morphology of the sepsis group revealed severe edema of the mucosal villi accompanied with severe intestinal gland injury, increased epithelial cell gaps, and severe hemorrhage. In the sepsis+MLD group, the putrescence and desquamation of epithelial cells in the intestinal mucosa were attenuated to some degree, whereas moderate mucosal sloughing still existed in villi tips (Figures 2(c) and 2(d)). The villi and glands were normal in both the control group and sham surgery group (Figures 2(a) and 2(b)). In the sepsis group, swollen hepatocytes and cytoplasmic lucency were observed. In addition, dilation of the sinus hepaticus and infiltration of lymphocytes in converged tube were also observed in the sepsis group. The degree of sinus hepaticus dilation and inflammatory cell infiltration was partially alleviated in the sepsis+MLD group (Figures 3(c) and 3(d)). There was no apparent change in the livers of the control group or sham surgery group (Figures 3(a) and 3(b)). In the sepsis+MLD group, the injury score data for intestine and liver only showed a downward trend (*p* > 0.05*vs.* sepsis group) (Figures 2(e) and 3(e)).

### 3.2. Effects of Sepsis and MLD on the mRNA Expression of TNF-*α*, IL-1*β*, and IL-6 in the Intestine, Liver, and Lung of Septic Rats

**Figure 4:**
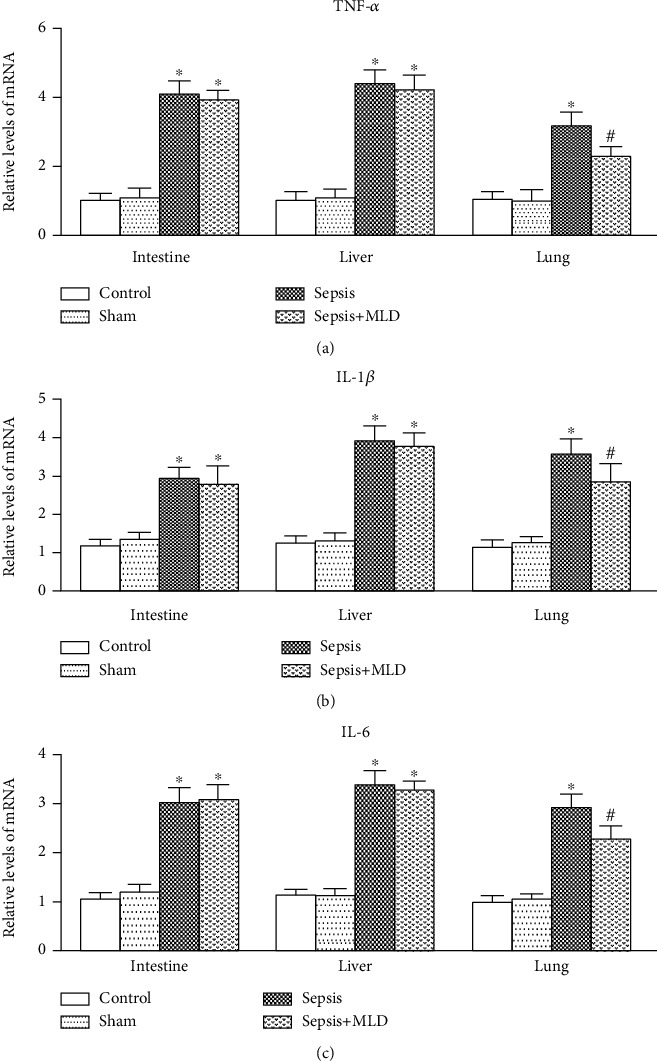
Expression of TNF-*α*, IL-1*β*, and IL-6 mRNA in intestine, liver, and lung tissues. Data were expressed as mean ± SD (*n* = 8). ^∗^*p* < 0.05 vs. control group, ^#^*p* < 0.05, sepsis+BTED group vs. sepsis group.

The expression levels of TNF-*α*, IL-1*β*, and IL-6 mRNA in the intestine, liver, and lung of the rats in the sepsis group increased significantly, compared with the control and sham surgery groups. In the sepsis+MLD group, the mRNA expression of TNF-*α*, IL-1*β*, and IL-6 in the lung was significantly decreased, compared with the sepsis group (*p* < 0.05). Additionally, the mRNA expression of TNF-*α*, IL-1*β*, and IL-6 in the intestine and liver showed a descending tendency in the sepsis+MLD group, but revealed no statistical significance, compared with the sepsis group (*p* > 0.05) (Figures 4(a)–4(c)).

### 3.3. Activity of Myeloperoxidase

**Figure 5:**
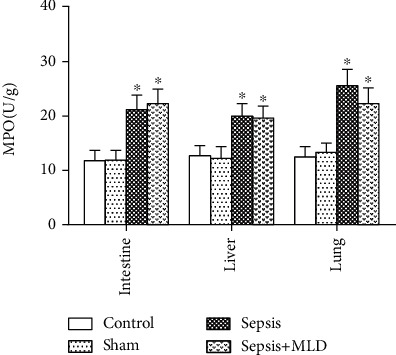
Effect of mesenteric lymph drainage on MPO levels of intestine, liver, and lung in septic rats. Results are presented as mean ± SD (*n* = 8).^∗^*p* < .01 vs. control group.

The MPO activity was used to assess the degree of inflammatory injury following sepsis. The levels of MPO in the lungs of rats in the sepsis group were significantly higher than those in the control and sham surgery groups, as shown in Figure 5 (*p* < 0.01). Compared with the sepsis group, the MPO levels in the lungs of the sepsis+MLD group showed an apparent downward trend (*p* > 0.05). However, the activity of MPO in the intestines and livers of the rats in the sepsis+MLD group revealed no evident changes, compared with the sepsis groups (*p* > 0.05).

It should be noted that, in our preliminary experiments, pulmonary inflammatory injury became apparent 6 hours after CASP surgery. Therefore, the results mentioned above were drawn from tissue specimens obtained 6 hours postoperatively.

### 3.4. TNF-*α*, IL-1*β*, and IL-6 Levels in Mesenteric Lymph and Serum

The levels of TNF-*α*, IL-1*β*, and IL-6 in septic mesenteric lymph increased significantly compared with normal lymph, normal serum, and even septic serum (Table 1).

### 3.5. Cocultivation of Primary PMVECs with Mesenteric Lymph

**Figure 6:**
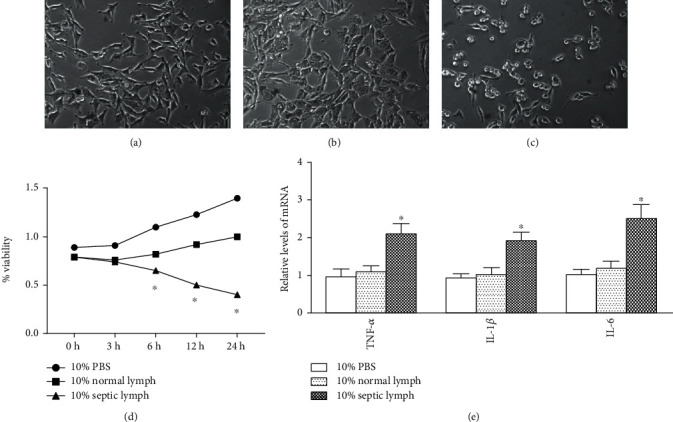
The morphological characteristics of PMVECs treated with mesenteric lymph (magnification, ×400). There was no morphologic change in PMVECs cocultivated with 10%PBS (a). When cocultured with 10% septic lymph, PMVECs showed shrinkage and detached from the bottom of the culture flask (c). In contrast, the injury of PMVECs was significant lessened when cocultured with 10% normal lymph (b). The cell viability of PMVECs was decreased by treatment with septic lymph for 24 h compared with that in the lymph group as measured by the MTT assay (d). TNF-*α*, IL-1*β*, and IL-6 mRNA expressions were significantly increased in PMVECs cocultivated with septic lymph compared with that in the normal lymph group (e). Results were analyzed using two-way repeated measures ANOVA with protected posthoc testing. ^∗^*p* < .01 vs.10% PBS group and 10% normal lymph.

After a 12-hour coincubation with 10% septic mesenteric lymph, most of the primary PMVECs shrunk, turned both smaller and rounder, and detached from the bottom of culture flask (Figure 6(c)). The morphological changes observed in the endothelial cells were significantly lessened with coincubated with normal lymph (Figures 6(a)–6(c)).

### 3.6. Assessment of Cell Viability and Detection of TNF-*α*, IL-1*β*, and IL-6 mRNA

MTT assay results indicated that the viability of PMVECs was significantly reduced by treatment with 10% septic lymph for 6, 12, and 24 h, compared with that of the PMVECs treated with normal lymph (all *p* < 0.05) (Figure 6(d)).

PMVECs treated with 10% septic lymph for 6 h exhibited much higher levels of TNF-*α*, IL-1*β*, and IL-6 mRNA than those in the group treated with 10% normal lymph or the control group (*p* < 0.01) (Figure 6(e)). There was no significant difference between the control and the normal lymph groups.

### 3.7. Analysis of PMVEC Apoptosis In Vitro

**Figure 7:**
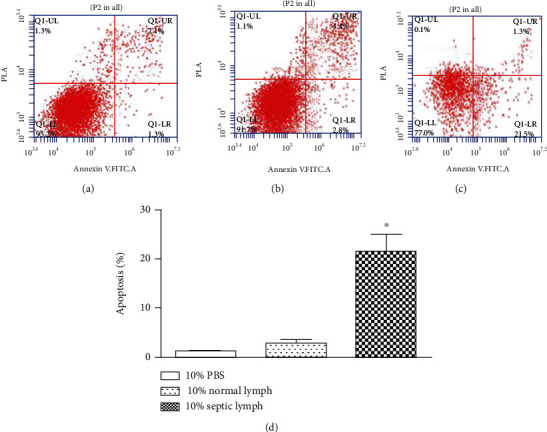
Flow cytometric analysis of PMVEC apoptosis using Annexin V-FITC/PI staining. The apoptosis rates of PMVECs were increased when treated with either septic lymph or normal lymph, compare with the control group. (a) Cocultivation of PMVECs with 10% PBS. (b) Cocultivation of PMVECs with 10% normal lymph. (c) Cocultivation of PMVECs with 10% septic lymph. (d) Data are presented as mean ± SD (*n* = 3), ^∗^*p* < .01 vs.10% PBS group and 10% normal lymph.

There was a significant increase (21.5% vs. 2.8%, *p* < 0.05) in the rate of apoptosis in the PMVECs treated with 10% septic lymph for 6 h when compared with the normal lymph control group (Figure 7). There was no significant difference between the control and normal lymph groups.

### 3.8. The Effect of Septic Mesenteric Lymph Duct Drainage on Pulmonary Endothelial Apoptosis and Permeability

**Figure 8:**
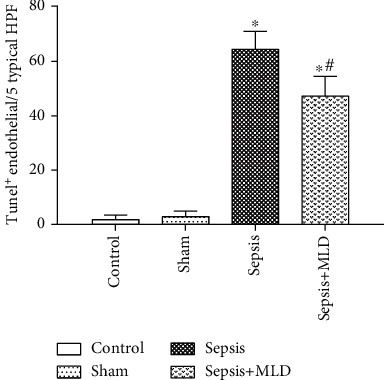
Effect of septic mesenteric lymph duct drainage on pulmonary endothelial apoptosis. Number of TUNEL-positive endothelial cells per 5 fields (hpf) in rats subjected to sham surgery, CASP sepsis model, and CASP+MLD. Data are shown as mean values ± SD (*n* = 8 per group). ^∗^*p* < .01 vs. control and sham surgery groups. ^#^*p* < .01 vs. sepsis group.

In the TUNEL assay, we observed an increase in the evidence of endothelial apoptosis in pulmonary tissues in the rats subjected to 6-hour CASP sepsis surgery, an effect that was not observed in the sham surgery group. We also found a significant increase in TUNEL-positive endothelial cells in the lungs of the sepsis group. This increase in pulmonary endothelial cell apoptosis induced by the CASP sepsis model was notably restrained by lymph duct drainage in the sepsis+MLD group (Figure 8). In addition, drainage of the mesenteric lymph partially prevented the increase in pulmonary permeability to Evans blue dye, when compared with the sepsis group. Pulmonary permeability was measured as the percentage of Evans Blue Dye (EBD) in bronchoalveolar lavage fluid (BALF) (Table 2).

## 4. Discussion

Initially, the gut has been referred to as origin of systemic inflammation/MODS due to the translocation of bacteria and their products from the gut into the systemic circulation [3, 24, 25]. Nevertheless, as research continues, it is believed that gut-derived factors not only migrate to other organs through the portal system but also diffuse into the gut-derived lymph to reach the systemic circulation via thoracic duct and potentially cause distant organs injury.

Recently, several manuscripts reported that lung injury is, at least in part, caused by tissue-injurious factors released from the ischemic gut and carried in the mesenteric lymph primarily through toxic effects upon pulmonary microvasculature and recruitment of neutrophils in experimental models of hemorrhagic shock or thermal injury [11, 26, 27]. This mode of lung inflammation and injury, caused by gut-derived inflammatory factors contained in the mesenteric lymph, has been named the “gut-lymph hypothesis of MODS” [12, 13, 28].

The gut-lymph hypothesis is based on 2 principal observations. First, that ligation of the mesenteric lymph duct was protective against lung injury [7, 28], as well as the systemic inflammation induced by trauma-hemorrhagic shock. Second, that in vitro neutrophil activation and endothelial cell dysfunction are caused by intestinal lymph from rats with trauma-hemorrhagic shock, but not sham shock [9–11]. The result we obtained from the histomorphology of lung tissues, expression of inflammatory cytokines, and activity of myeloperoxidase revealed that mesenteric lymph drainage could alleviate inflammatory injury in the pulmonary tissue of septic rats, when compared with tissues from the intestine and liver. Our in vitro experiments also revealed that, in PMVECs cocultured with septic mesenteric lymph, the expression of inflammatory mediators, pathologic morphology, and cell viability were obviously abnormal when compared to PMVECs treated with normal lymph, lending support to the conclusions drawn from the in vivo studies. Our study provides potent evidence for the implication of the “gut-lymph hypothesis” in ALI in sepsis.

Mesenteric lymph avoids the portal circulation and thus bypasses the reticuloendothelial system in the liver, i.e., the secondary firewall mediated by KCs. Any unfiltered luminal constituents, such as endotoxins, locally produced cytokines and activated immune cells that exit the MLN, are able to directly leak to the circulation. In our study, we demonstrated that septic mesenteric lymph from the intestines of CASP septic rats contained higher levels of the proinflammatory mediators, TNF-*α*, IL-1*β*, and IL-6. In the present sepsis model, the first organ to demonstrate the inflammatory response was the gut, which is analogous to the results of our previous studies in rats with hemorrhagic shock [19] (data not shown). According to the preceding analysis, we concluded that the gut transduces intestinal septic insult into an injurious pulmonary inflammatory response through the production of gut-derived inflammatory mediators, like TNF-*α*, IL-1*β*, and IL-6, that reach the systemic circulation via the mesenteric lymph. It is uncertain which mediators are responsible for the effects that the lymph from hemorrhagic shock has on endothelial cells. Nevertheless, the presence of TNF, IL-1, IL-6, and PLA_2_ in the mesenteric lymph from posthemorrhagic shock is correlated to the activation of circulating neutrophils and subsequent lung injury [29]. Moreover, the apparent increase of IL-6 and sTNF-R_55_ in the thoracic duct lymph was confirmed in MODS patients [30]. Furthermore, pentoxifylline, an inhibitor of TNF-*α*, can alleviate lung injury by decreasing the content of TNF-*α* in the serum and mesenteric lymph following ischemia/reperfusion (I/R) injury [31]. In our sepsis model, the injurious pulmonary inflammatory response is closely related to high levels of inflammatory mediators (TNF-*α*, IL-1*β*, and IL-6) found in the septic mesenteric lymph, especially with respect to the effect of the amplified inflammatory cascade response seen in septic syndrome. We therefore reasoned that inflammatory mediators, such as TNF-*α*, IL-1*β*, and IL-6, contained within the septic mesenteric lymph play an indispensable role in the lung injury induced by sepsis.

Both animal and clinical studies have documented that ALI and ARDS are associated with increased pulmonary cellular apoptosis [32–34]. Nonetheless, these studies have largely focused on the pulmonary epithelial cell population [33, 35, 36]. In indirect ALI, such as nonpulmonary sepsis, the injury-inducing factors reach the lung via the systemic circulation, with the endothelium being the first pulmonary cell population exposed to these factors. In this article, we documented that apoptosis of PMVECs was induced dramatically by septic mesenteric lymph. Simultaneously, in our in vivo experiments, there was a significantly higher number of apoptotic endothelial cells in the lungs of the sepsis group, which was negated by mesenteric lymph duct drainage. In the trauma-hemorrhagic shock model, it appears that mesenteric lymph-induced endothelial cell apoptosis involves both caspase-dependent and caspase-independent pathways [32, 37]; however, the mechanism of septic lymph-induced endothelial cell apoptosis has not been reported. It has been found that, during the inflammatory response, many proinflammatory mediators such as TNF-*α*, IL-1*β*, and IL-6 moderate the activation of hypoxia inducible factor-1 alpha (HIF-1a), which is closely involved in regulating mitochondrial-dependent cell death [38, 39]. Since the essence of sepsis is the process of cascade amplification of inflammatory responses, probing the relationship between inflammatory injury and apoptosis of pulmonary endothelial cells is a vital goal for subsequent research on the topic of sepsis.

We found that the lungs in the sepsis group showed significant leakage of Evans Blue 6 h following induction of sepsis, indicating an increased disruption of the endothelial barrier in this group, compared to the sepsis+MLD group. There are many similarities between endothelial cell apoptosis and increased endothelial permeability, both of which appear to occur in acute respiratory distress syndrome following shock and sepsis, an interesting point of functional significance when considering the apoptosis of pulmonary endothelial cells [40].

Unlike previous works, we used rat primary pulmonary microvascular endothelial cells to investigate the potential cytotoxicity of endothelial cells involved in septic lymph, which was a reasonable course of action both clinically and biologically, when considering the microvascular vulnerability and permeability changes during pulmonary interstitial edema.

Even by breaking the gut–lymph–lung axis, the inflammatory injury of lung could be attenuated. Other mechanism like gut–liver crosstalk also takes part in systemic inflammation and lung injury in sepsis [41–44]. Unlike the biliary tract external drainage by percutaneous transhepatic catheter drainage (PTCD) or endoscopic nasobiliary drainage (ENBD), mesenteric lymph drainage is hard to mimic in clinical practice. How to mediate the toxic effects of mesenteric lymph? It appears to be determined by the free fatty acids- (FFA-) to-protein ratio in mesenteric lymph, as the addition of albumin-a lipid scavenger-reversed their effects [45]. Mice with genetic mutations in the receptors or adapter molecules from the TLR4 signaling pathway were protected against postshock mesenteric lymph-mediated lung injury [46]. Basic insights into the intimate relationship between the gut–lymph–lung axis and the systemic inflammatory system are expected to lead to more efficacious treatment modalities for sepsis in the future.

In summary, the results of this study demonstrated that mesenteric lymph duct drainage is able to alleviate pulmonary inflammatory injury as measured by histomorphology of lung tissues, expression of inflammatory cytokine, and activity of myeloperoxidase, and that endothelial cells apoptosis mediated by septic mesenteric lymph plays a momentous role in the dysfunction of pulmonary microvascular permeability in septic rats. These findings suggest an important role for the “gut-lymph hypothesis” in sepsis-induced ALI and may provide the basis for novel therapeutic approaches to MODS.

## 5. Conclusions

Mesenteric lymph duct drainage can significantly attenuate pulmonary inflammatory injury in septic rats, by decreasing the expression of pivotal inflammatory mediators and inhibiting apoptosis, to preserve the function of the pulmonary endothelial barrier.

## Figures and Tables

**Table 1:** TNF-*α*, IL-1*β*, and IL-6 levels in normal/septic mesenteric lymph and serum.

	Normal serum	Septic serum	Normal lymph	Septic lymph
TNF-*α*	20.88 ± 7.85	95.92 ± 16.03^∗^	75.26 ± 11.25	176.52 ± 19.72^∗^^#^
IL-1*β*	43.95 ± 4.84	67.38 ± 3.82^∗^	55.67 ± 7.25	104.76 ± 10.42^∗^^#^
IL-6	306.25 ± 13.44	850.82 ± 37.45^∗^	442.61 ± 18.94	1398.73 ± 43.25^∗^^#^

Data are expressed as mean ± SD, *n* = 8 per group, ^∗^*p* < .05 versus normal serum/lymph #*p* < .05 versus septic serum.

**Table 2:** Effect of mesenteric lymph drainage on lung permeability in CASP sepsis rats.

Group	Lung permeability (% EBD in BALF)
Control	3.20 ± 0.37
Sham surgery	3.23 ± 0.40
Sepsis	11.21 ± 1.47^∗^
Sepsis+MLD	9.09 ± 0.95^∗^^#^

Data are expressed as mean ± SD, *n* = 8 per group, ^∗^*p* < .01 vs.control/sham group, #*p* < .05 vs. sepsis group.

## Data Availability

The data used to support the findings of this study are available from the corresponding author upon request.
